# The Carnosine–HNE Michael Adduct as a Redox-Active Species Associated with Nrf2-Dependent Antioxidant and Anti-Inflammatory Responses

**DOI:** 10.3390/antiox15030388

**Published:** 2026-03-19

**Authors:** Alessandra Altomare, Giovanna Baron, Francesca Gado, Larissa Della Vedova, Giulio Ferrario, Lara Davani, Ettore Gilardoni, Rebecca Ferrisi, Clara Mocchetti, Lavpreet Singh, Barbora De Courten, Marina Carini, Rosalba Siracusa, Ramona D’Amico, Rosanna Di Paola, Clelia Dallanoce, Daniela Impellizzeri, Giancarlo Aldini

**Affiliations:** 1 Department of Pharmaceutical Sciences, University of Milan, Via Mangiagalli 25, 20133 Milan, Italy; alessandra.altomare@unimi.it (A.A.); giovanna.baron@unimi.it (G.B.); francesca.gado@unipv.it (F.G.); l.dellavedova@uu.nl (L.D.V.); lara.davani@uniurb.it (L.D.); ettore.gilardoni@philochem.ch (E.G.); rebecca.ferrisi@unimi.it (R.F.); clara.mocchetti@unimi.it (C.M.);; 2 School of Health and Biomedical Sciences, Royal Melbourne Institute of Technology University, Melbourne 3000, Australia; 3 Department of Chemical, Biological, Pharmaceutical and Environmental Science, University of Messina, 98122 Messina, Italy; 4 Department of Medicine and Surgery, “Kore” University of Enna (UKE), 94100 Enna, Italy; 5 Department of Veterinary Sciences, University of Messina, 98168 Messina, Italy

**Keywords:** carnosine, 4-hydroxy-2-nonenal (HNE), Nrf2 activation, NF-κB, anti-inflammatory, antioxidant

## Abstract

Carnosine (CAR), an endogenous histidine-containing dipeptide, exhibits antioxidant and anti-inflammatory activity in various experimental models; however, its molecular mechanism of action remains poorly understood. Here, we demonstrate that the Michael adduct between CAR and 4-hydroxy-2-nonenal (HNE), which has been detected in previous studies in both in vitro and in vivo settings, mediates its bioactivity, particularly its antioxidant and anti-inflammatory responses, through Nrf2 activation. The CAR–HNE adduct was synthesized and its physicochemical, metabolic, and biological properties were evaluated. CAR–HNE exhibited high stability in biological matrices and retained the ability to transfer HNE to thiol nucleophiles at a slow rate under physiologically relevant conditions, consistent with electrophile-mediated Nrf2 activation. This kinetic behavior limits the cytotoxicity typically associated with free HNE while preserving the redox signaling capacity. CAR–HNE induced dose-dependent Nrf2 activation and NF-κB inhibition in cell-based assays without the hormetic toxicity observed for free HNE. Mechanistically, CAR–HNE may act as a redox-tunable electrophilic reservoir, restoring nucleophilic tone and modulating redox-sensitive transcription factors. In vivo, CAR–HNE attenuated DSS-induced colitis more effectively than equimolar doses of either carnosine or HNE alone. Proteomic analyses revealed modulation of canonical Nrf2-dependent antioxidant pathways. Our findings suggest a conceptual shift in carnosine biology: rather than acting as a classical antioxidant or carbonyl quencher, carnosine functions as a precursor of redox-active electrophilic adducts that transduce anti-inflammatory and antioxidant responses via controlled RCS signaling.

## 1. Introduction

### 1.1. Carnosine as an Antioxidant and Anti-Inflammatory Peptide

Carnosine (β-alanyl-L-histidine) (CAR) is an endogenous dipeptide that contributes to the regulation of oxidative stress and inflammatory responses, as demonstrated by in vitro and in vivo studies summarized in recent reviews [1,2,3]. Genetic models manipulating tissue CAR levels have provided further evidence of its antioxidant function: overexpression of CAR-synthesizing enzymes reduces oxidative stress, whereas depletion leads to its exacerbation [4,5].

The molecular basis of CAR’s antioxidant activity remains only partially understood. CAR may act both directly, by neutralizing reactive species, and indirectly, under specific conditions, by upregulating antioxidant defense genes through activation of the transcription factor nuclear factor erythroid 2-related factor 2 (Nrf2) [1]. In vitro, CAR acts at multiple stages of the oxidative cascade, including transition metal chelation, scavenging of reactive oxygen species (ROS) and peroxyl radicals, and protection against hypochlorous acid (HOCl) [6].

However, the physiological relevance of its direct antioxidant action is questionable. Under in vivo conditions, CAR cannot effectively compete with enzymatic antioxidants such as catalase, superoxide dismutase, and glutathione peroxidase, which are far more efficient at detoxifying hydrogen peroxide (H_2_O_2_) and superoxide anions. Although CAR has been shown to scavenge hydroxyl radicals in vitro [7], this reaction is unlikely to be significant in vivo, as hydroxyl radicals react almost instantaneously with a wide range of biomolecules [7]. The reaction rate constants of CAR with oxidants are also lower than those of thiol-containing peptides and proteins. For example, the initial reaction rate of CAR and its derivatives with HOCl ranges from 0.5 to 1.6 × 10^5^ M^−1^ s^−1^ [8,9], significantly lower than the rates observed for HOCl reactions with methionine and cysteine (10^7^–10^8^ M^−1^ s^−1^) [10].

CAR and other histidine-containing dipeptides, such as anserine and homocarnosine, have been found to undergo oxidation in vivo, as confirmed by the identification of their oxidized derivatives (2-oxo-carnosine, 2-oxo-anserine, and 2-oxo-homocarnosine) [11]. Therefore, rather than acting as antioxidants per se, histidine dipeptides appear to function as oxidizable substrates. This aligns with Halliwell’s definition of an antioxidant as “any substance that, when present at low concentrations compared with those of an oxidizable substrate, significantly delays or prevents oxidation of that substrate” [12]. Given the relatively low reaction constants of CAR with oxidants and its tissue concentrations, it is unlikely to effectively compete with enzymatic antioxidants for most oxidizing agents or with cellular substrates for radical scavenging. However, in specific tissues, such as skeletal muscle, where CAR is present at millimolar concentrations, its high local abundance may increase the reaction rates according to the second-order rate equation: v = k[CAR][oxidants]

### 1.2. Carnosine as a Sequestering Agent of Reactive Carbonyl Species (RCS)

Research has demonstrated that CAR effectively traps several aldehydes derived from lipid and sugar oxidation, particularly α,β-unsaturated aldehydes, via a multistep reaction involving both its primary amine and imidazole rings. This reactivity involves both the β-alanine amino group and the histidine imidazole ring, resulting in the formation of stable Michael adducts [13]. The interaction of CAR with α-(glyoxal, GO; methylglyoxal, MGO) and β-(malondialdehyde, MDA) dicarbonyls has also been investigated and their reaction products characterized [14]. The findings suggest that CAR exhibits moderate reactivity toward MDA, forming N-propenal adducts via Michael addition, while its quenching activity toward GO and MGO is relatively poor, leading to the formation of GOLD- and MOLD-like adducts through a concerted reaction involving multiple quencher molecules [15].

Additionally, CAR has been shown to detoxify catechol aldehydes—highly reactive electrophiles generated by the oxidative deamination of norepinephrine and dopamine via monoamine oxidase. These aldehydes are implicated in the pathogenesis of neurodegenerative diseases and ischemic and diabetic cardiac injuries [16].

While CAR–RCS adducts have been well characterized in vitro [15], their in vivo occurrence has been confirmed primarily for adducts with α,β-unsaturated aldehydes, such as acrolein (ACR) [17,18,19,20] and 4-hydroxynonenal (HNE) [19,21], due to their higher reactivity and selectivity toward CAR.

Among the CAR–RCS adducts, the Michael adduct formed between CAR (CAR) and 4-hydroxy-2-nonenal (HNE), namely CAR–HNE, has been the most extensively investigated by our group and others. We initially performed a comprehensive in vitro structural characterization of the CAR–HNE adduct using NMR and mass spectrometry analyses [22], and this structural assignment was subsequently independently confirmed by the group of Sayre [23]. We then identified the adduct in biological matrices: first, in vitro tissue preparations such as skeletal muscle [24], and subsequently, in ex vivo samples. Using a validated LC–MS approach, CAR–HNE was detected in the urine of Zucker rats and proposed as a biomarker of carbonylation damage [25]. The in vivo formation of CAR–HNE adducts was later confirmed by additional independent studies in both animal models and human samples [17,19,20]. Notably, a significant increase in CAR–HNE levels, together with GS–HNE, was observed in skeletal muscle tissue of end-stage amyotrophic lateral sclerosis models, further supporting the biological relevance of this conjugation pathway under conditions of oxidative stress [21].

The concept that CAR’s biological effects stem from RCS detoxification is supported by a growing body of experimental data [1]. However, this mechanism is unlikely to be quantitatively dominant in vivo, as CAR’s reaction rates are markedly lower than those of major cellular nucleophiles. The rate constant for CAR–HNE adduct formation (k = 0.035 M^−1^ s^−1^) [26] is orders of magnitude lower than for cellular (GSH, k = 3.81 M^−1^ s^−1^) and extracellular (HSA-Cys34, k = 50.6 M^−1^ s^−1^) nucleophiles. Consequently, CAR is expected to react with a fraction of HNE relative to major thiol nucleophiles. However, the consistent in vivo detection of CAR–HNE indicates that this reaction, although kinetically less favored, occurs at measurable levels under physiological and pathological conditions.

Nevertheless, in vivo CAR administration has been consistently associated with reduced protein carbonylation and lower RCS-mediated damage [4,27,28,29]. This suggests that CAR may exert an RCS-detoxifying effect through an alternative, indirect mechanism rather than a direct scavenging action. The presence of HNE and ACR adducts with CAR in in vivo studies indicates that this reaction occurs, albeit with limited efficiency unless CAR levels are high and GSH levels are relatively low.

Collectively, these findings establish that CAR–HNE formation is not merely a chemical possibility observed in vitro but a reproducible in vivo event occurring under conditions of enhanced lipid peroxidation.

### 1.3. Carnosine as an Nrf2 Activator

Recent studies suggest a novel role for CAR as an activator of the nuclear factor erythroid 2-related factor 2 (Nrf2) [30,31,32,33]. This discovery provides a potential explanation for CAR’s antioxidant effects and its ability to reduce RCS-induced protein carbonylation. Nrf2 activation upregulates enzymes involved in RCS detoxification, including aldose reductase, oxidases, and glutathione transferases. However, Nrf2 activation classically requires electrophilic species capable of covalently modifying key cysteine residues on Keap1. As CAR is a nucleophilic peptide rather than an electrophile, direct activation of Nrf2 through the canonical Keap1–cysteine modification mechanism appears chemically unlikely.

### 1.4. Reactive Carbonyl Species as Nrf2 Activators and Their Hormetic Response

Reactive carbonyl species themselves are established electrophilic activators of Nrf2 [34]. Due to their high reactivity, RCS act locally, exerting paracrine effects rather than diffusing over long distances. At low concentrations, RCS—particularly HNE—exhibit hormetic behavior, promoting adaptive stress responses through the activation of Nrf2 and other redox-sensitive signaling pathways, such as tyrosine kinase receptors. These adaptive effects contribute to enhanced antioxidant defenses, improved metabolism, and cellular survival. At higher concentrations, however, RCS induce cytotoxicity, oxidative damage, and inflammation [35,36,37].

### 1.5. Rationale and Aim of the Study

Taken together, these findings define CAR as a bioactive molecule whose antioxidant and anti-inflammatory properties cannot be fully explained by direct radical scavenging or simple RCS quenching. Given the established in vivo formation of CAR–RCS adducts, particularly CAR–HNE, we sought to investigate whether these conjugates possess intrinsic biological activity beyond passive detoxification.

We hypothesize that the stable Michael adduct formed between CAR and HNE (CAR–HNE) may act as a persistent electrophile-bearing species capable of modulating redox-sensitive signaling pathways. Rather than functioning as a freely diffusible aldehyde, CAR–HNE may represent a chemically stabilized form of HNE with distinct kinetic and biological properties. Through this mechanism, transient reactive aldehydes could be converted into more stable conjugates capable of sustaining low-level electrophilic signaling, including Nrf2 activation, without eliciting overt cytotoxicity.

In this study, we therefore investigated the molecular stability, thiol reactivity, and biological activity of CAR–HNE, with a particular focus on its antioxidant and anti-inflammatory effects.

## 2. Materials and Methods

### 2.1. Synthesis of CAR–HNE

All solvents were obtained from Sigma-Aldrich Srl (Milan, Italy) and used as supplied, without additional purification. The 1H-NMR spectrum has been registered with a Varian Mercury 300 instrument (300 MHz, Varian, Inc., Palo Alto, CA, USA). The reaction was monitored using thin-layer chromatography (TLC) on commercially available aluminum plates coated with silica gel 60 (F-254, Merck, Milan, Italy); spots were further evidenced by spraying with Ninhydrin solution. The high-resolution mass spectrum was acquired with an LTQ Orbitrap XL mass spectrometer (Thermo Scientific, Milan, Italy) equipped with an ESI source acquiring in positive ion mode. The source parameters were set as follows: spray voltage 4.0 kV, capillary temperature 275 °C, sheath gas (nitrogen) 10 a.u., capillary voltage 42 V, tube lens offset 120 V. Full MS spectra were acquired in profile mode by the FT analyzer in a scan range of *m*/*z* 100–600, using a resolution of 30,000 FWHM at *m*/*z* 400. Compounds were dissolved in Milli-Q H_2_O (purified at 18 MΩcm by a purification system produced by Millipore, Bedford, MA, USA) and appropriately diluted with CH_3_CN/H_2_O/HCOOH 50:50:0.1 (%*v*/*v*) before direct infusion into the mass spectrometer.

### 2.2. CAR–HNE Dissociation Constant and HNE Transfer

CAR–HNE constant reactions: To characterize the kinetics and equilibrium of the reversible reaction between CAR and HNE, the time-dependent concentration of HNE was monitored by HPLC-UV [27] under controlled conditions. This approach allowed determination of the association rate constant (kon), the dissociation rate constant (koff), and the equilibrium constant. To determine the kon, CAR (1 mM) was mixed with HNE (30 µM) so that the concentration of CAR remained effectively constant. Under these pseudo-first-order conditions with respect to HNE, the decay of free HNE as a function of the time was fitted to a single-exponential model to obtain the kon. To determine the koff, a pre-formed CAR–HNE adduct (200 µM) was allowed to dissociate in phosphate buffer (PBS) starting in the absence of free reactants. The appearance of HNE (equivalently, the disappearance of CAR–HNE) was monitored over time and fitted with a first-order model to obtain the koff. Finally, the equilibrium constant was calculated as the ratio of the kon to the koff.

HNE transfer to GSH: The trans-Michael transfer of HNE from CAR–HNE to GSH was measured by incubating CAR–HNE with glutathione in a 1:100 molar ratio at pH 6.4 (obtained with 1 mM PBS) or 7.4 (obtained with 1 mM PBS) and 8.4 (obtained with 1 mM NH_4_HCO_3_) at 37 °C for 90 min. After incubation, samples were diluted in 80% of acetonitrile (ACN), 20% of water and 0.1% formic acid (FA) and injected via direct infusion into an LTQ-XL Orbitrap. The ion source parameters were set as follows: spray voltage 3.5 kV; capillary temperature 275 °C; sheath gas 5 units. The Orbitrap mass spectrometer was operated in positive mode with a resolution of 100,000 (FWHM at 400 *m*/*z*), AGC of 3 × 10^6^, and a maximum injection time of 250 ms. The formation of the GSH–HNE conjugate was monitored over time (90 min) by detecting the [M + H]^+^ ion at *m*/*z* 464.20111.

### 2.3. Metabolic Stability Studies

Blank rat tissues were obtained from Accelera (Nerviano, Milan, Italy). The animals were handled in accordance with applicable institutional and national guidelines for the care and use of laboratory animals. Tissues were homogenized in PBS 1 mM pH 7.4 at 4 °C to have a final concentration of 0.1 mg/mL of wet tissue. Aliquots of the homogenized tissue or human serum (Sigma-Aldrich Srl, Milan, Italy) were pre-incubated at 37 °C for 10 min, after which a CAR–HNE stock solution was spiked into the samples to reach a final concentration of 5 µM. At 0, 5, 15, 30, 60, and 120 min following CAR–HNE addition, samples were deproteinized by adding 10 volumes of ACN and kept at 4 °C for 10 min. The mixtures were then centrifuged at 14,000 rpm at 4 °C per 10 min. Finally, 10 µL of supernatant was directly injected into the LC–MS system for the analysis. Chromatographic separation was performed on an Exion LC 100 system (AB Sciex, Milan, Italy) with a Thermo Scientific Hypersil GOLD HILIC column (150 × 2.1 mm, 3 µm particle size, 175 Å pore size, Thermo Fisher, Milan, Italy) maintained at 40 °C. The flow rate was set at 250 µL/min with acetonitrile as mobile phase A and an aqueous solution of ammonium formate (100 mM, pH corrected to 3 with formic acid), as mobile phase B. Elution was achieved with a binary gradient from 5% to 70% B over 6.5 min, held at 70% B for 3 min, then re-equilibrated at 5% B for 6 min. The column effluent was directly introduced into an API 4000 mass spectrometer (AB Sciex, Milan, Italy) equipped with a TurboV electrospray interface (AB Sciex, Milan, Italy). The electrospray ionization was carried out in positive ion mode by applying +3.5 kV ionization potential, 25 units of curtain gas, 40 units of gas 1, and 60 units of gas 2 heated at 550 °C. Detection was performed in multiple reaction monitoring (MRM) mode, monitoring the transitions *m*/*z* 383.20 → 312.20 (collision energy 32 V) and *m*/*z* 383.20 → 266.60 (collision energy 34 V). Quantification was based on the peak area of the 383.20 → 312.20 transition. The signal intensity at each time point was normalized to the mean peak area at 0 min and expressed as the residual percentage of CAR–HNE remaining in the sample.

### 2.4. Cell-Based Assays

#### 2.4.1. Cell Viability

Cell viability was evaluated using the luminescent CytoTox-Glo™ Cytotoxicity Assay (Promega, Madison, WI, USA), which quantifies the activity of a dead-cell protease released upon loss of membrane integrity. The assay was performed on Nrf2/ARE Responsive Luciferase Reporter HEK293 stable cells (Signosis, Santa Clara, CA, USA), seeded at a density of 10,000 cells per well in white 96-well plates. The cells were treated under the experimental conditions described in the following: Section 2.4.2 and Section 2.4.4. At the end of the treatment, the CytoTox-Glo™ reagent was added according to the manufacturer’s protocol, and the luminescence was measured after brief incubation at room temperature using a microplate reader (Synergy 2, BioTek^®^ Instruments, Inc., Highland Park, VT, USA). As a positive control for complete cell lysis, an equivalent number of untreated cells were sonicated in a water bath for 10 min at 37 °C to generate total cell lysates. The luminescence values were first normalized by subtracting the background signal (medium only), and the results were expressed as fold-change in luminescence relative to untreated control cells. The experiments were conducted with both biological and technical replicates, and the results are expressed as the mean ± SD relative to untreated control cells. Statistical significance was determined using one-way ANOVA followed by Bonferroni’s multiple comparisons test, with *p* < 0.05 considered significant.

#### 2.4.2. Nrf2 Gene Reporter Cell Model

CAR–HNE was analyzed for its potential to influence the cell antioxidant response through Nrf2 activation, utilizing the Nrf2/ARE Responsive Luciferase Reporter HEK293 stable cell line (Signosis, Santa Clara, CA, USA), as previously reported [38]. In brief, HEK293 cells (10,000 cells per well) were exposed to CAR–HNE, CAR, or HNE at concentrations ranging from 1 to 50 µM. Bardoxolone methyl (CDDO-Me) at 75 nM was used as a positive control [39]. After adding ONE-Glo™ Luciferase Assay Substrate (Promega Corporation, Madison, WI, USA) (100 µL per well), luminescence was detected using a Synergy 2, BioTek^®^ Instruments, Inc., Highland Park, VT, USA. The experiments were conducted with both biological and technical replicates, and the results are expressed as the mean ± SD relative to untreated control cells. Statistical significance was determined using one-way ANOVA followed by Bonferroni’s multiple comparisons test, with *p* < 0.05 considered significant.

#### 2.4.3. PathHunter^®^ Enzyme Fragment Complementation Assay Platform

The dose-dependent Keap1-Nrf2 pathway activation by CAR–HNE and HNE via nuclear translocation of Nrf2 was tested using the PathHunter^®^ eXpress Keap1-Nrf2 Nuclear Translocation Assay (Eurofins, Discover X, Fremont, CA, USA) according to the manufacturer’s instruction.

#### 2.4.4. NF-κB Gene Reporter Cell Model

The in vitro anti-inflammatory activity of CAR–HNE was assessed using a previously described cell model [38]. Briefly, HEK293 NF-κB cells were seeded in a 96-well blank plate (BRANDplates^®^, cell grade) at a density of 15,000 cells/well, and after 6 h, the cells were pre-treated for 18 h with varying concentrations of CAR–HNE, CAR, or HNE (1–50 µM) in complete medium (MEM, 10% FBS, 1% NEAA, 1 mM Na pyruvate, 1% penicillin/streptomycin). A 25 µM concentration of 5-(3′,4′-dihydroxyphenyl)-γ-valerolactone (VL) was used as a positive control [40]. Following pre-treatment, the cells were stimulated with 0.01 ng/mL IL-1α for 6 h. After incubation, the medium was removed and replaced with 50 µL MEM per well. Luciferase activity was measured using a luminometer (Synergy 2, BioTek^®^ Instruments, Inc., Highland Park, VT, USA) after adding 50 µL of ONE-Glo™ Luciferase Assay Substrate (Promega Corporation, Madison, WI, USA). The experiments were conducted with both biological and technical replicates, and the results are reported as the mean ± SD relative to untreated control cells. Statistical analysis was performed using one-way ANOVA followed by Bonferroni’s multiple comparisons test, with *p* < 0.05 considered statistically significant.

### 2.5. In Vivo Studies

#### 2.5.1. Dextran Sodium Sulfate (DSS)-Induced Colitis

##### Animals

C57BL/6J male mice (25–30 g) (aged 10–12 weeks) were obtained from Envigo (Milan, Italy) and housed in a sterile environment with water and food suitable for mice. The cages in which the animals were kept were in rooms with a temperature of 22 ± 1 °C and day/night cycles of 12 h each. They were kept in a controlled location and received food and water ad libitum. The review board for the care of animals at the University of Messina approved the study (approval number: n. 1148/2024-PR, approval date 17 December 2024). All animal experiments accorded with the new Italian regulations (D.Lgs 2014/26) and EU regulations (EU Directive 2010/63) (approval number 1148/2024-PR).

##### Colitis Induction

To induce colitis, the animals were administered with DSS 3% in drinking water for 7 days followed by a 3-day recovery period with regular drinking water [41,42]. After 10 days, the animals were sacrificed, with the abdomen opened by a midline cut. The colon was removed and freed from surrounding tissues, opened along the antimesenteric border, washed, weighed and processed for histological and biochemical studies.

##### Experimental Design

The animals were randomly divided into several groups (n = 6 for each group for each technique), where the total number of animals for each experimental group = 18 (Figure S1, Supplementary Data). The sample size analysis was conducted by G. Power 3.1, respectively, ANOVA (fixed effects, omnibus, one-way) “a priori”, Effect size (f) = 0.25, a significance level a = 0.05, a power approximately (1 − β) = 0.95.

1. Sham + vehicle group (SHAM): Vehicle alone (water) was administered in the control (sham) group instead of DSS.
2. DSS + vehicle group (DSS): Mice were subjected to DSS administration as described above, and saline was administered by oral gavage from 3rd day until 10th day.
3. DSS + HNE (5.77 mg/kg) group (DSS_HNE): Mice were subjected to DSS administration as described above, and HNE (5.77 mg/kg) was administered by oral gavage from 3rd day until 10th day.
4. DSS + CAR (8.36 mg/kg) group (DSS_Carnosine): Mice were subjected to DSS administration as described above, and CAR (8.36 mg/kg) was administered by oral gavage from 3rd day until 10th day.
5. DSS + CAR–HNE (14.5 mg/kg) group (DSS_CAR–HNE): Mice were subjected to DSS administration as described above, and CAR–HNE (14.5 mg/kg) was administered by oral gavage from 3rd day until 10th day.

Since no significant change was found between the sham groups (Sham + CAR, Sham + HNE, Sham + CAR–HNE), we presented data of the sham + vehicle group only. The doses of HNE, CAR and CAR–HNE were chosen based on previous studies [31,43].

For randomization, we assigned identification numbers to each animal and then used a basic online random number generator that generated random numbers and allocated animals to study groups. Each cage was numbered and labeled according to its assigned treatment, ensuring clear identification while preventing systematic bias in handling. The investigators performing the analysis and data quantification were blinded to the group allocation. Group identity was revealed only after completion of the data analysis.

No specific analgesic or anesthetic interventions were required for the experimental procedures. The animals were monitored daily for signs of pain, suffering, or distress, and they were maintained under standard housing conditions throughout the study.

##### Body Weight, Colon Length and Macroscopic Analysis of Colon Damage

The mice were weighed from day 0 until the day of sacrifice. The colon length was measured as previously described by Cordaro et al. [41]. Briefly, the colon was excised between the ileocecal junction and the proximal rectum, close to its passage under the pelvisternum, and was measured with a standard ruler. The total colon was washed with saline, opened by a longitudinal incision, and examined under a microscope. Macroscopic damage was assessed as previously described by Elsheikh et al. [44].

##### Histological Examination

For the histological analysis, tissues were collected, fixed in 10% of buffered formalin phosphate, embedded in paraffin, sectioned and subjected to hematoxylin and eosin staining. The degree of histological damage of the colon sections was scored based on previous work by Cordaro et al. [41]. Five H&E stained sections from each mouse were scored in a blinded fashion, using a Leica DM6 microscope (Leica Microsystems SpA, Milan, Italy) associated with Leica LAS X Navigator software (v. 3.4.2.1836.8.1.2 Leica Microsystems SpA, Milan, Italy).

##### Myeloperoxidase Assay

MPO activity, an indicator of neutrophil infiltration, was determined by spectrophotometric assay with tetramethylbenzidine as the substrate. The collected colons were homogenized in a solution containing 0.5% hexadecyl-trimethyl-ammonium bromide dissolved in potassium phosphate buffer (10 mM pH 7) and subsequently centrifuged for 30 min at 20,000× *g* at 4 °C. Part of the supernatant was then allowed to react with a solution of tetramethylbenzidine (1.6 mM) and H_2_O_2_ (0.1 mM). A reading at 650 nm was performed using a spectrophotometer. MPO activity was expressed in units of MPO/g of wet tissue and was measured as the quantity of enzyme degrading 1 µM of peroxide per min at 37 °C [45,46].

#### 2.5.2. Quantitative Proteomic Studies

Colon tissue samples from three of the above-mentioned groups, SHAM, DSS and DSS + CAR–HNE, were carefully collected and stored for proteomic studies. For protein digestion, we implemented an optimized solid-phase protocol using disposable S-TRAP devices (ProtiFi, Huntington, New York, NY, USA), which significantly improved the enzymatic digestion efficiency and peptide recovery. Proteomic analysis was performed using nano-LC-HRMS (nano Liquid Chromatography-High-Resolution Mass Spectrometry) on a Dionex Ultimate 3000 nano-LC system (Thermo Scientific, Sunnyvale, CA, USA) coupled to an Orbitrap Fusion Tribrid mass spectrometer (Thermo Scientific, Bremen, Germany). The resulting proteomic data were processed with MaxQuant v.1.6.0, while Perseus v.1.6.1.43 was used for the statistical analysis. To identify differentially regulated proteins and modulated pathways across experimental conditions, functional analysis tools were applied, including STRING v.1.7.0 and Ingenuity Pathway Analysis (IPA v.153384343).

### 2.6. Statistical Analysis

All the values are expressed as the mean ± standard error of the mean of N observations. For the in vivo experiments, N represents the number of animals. For the experiments involving histology, the photos shown are demonstrative of at least three experiments performed on different experimental days on tissue sections collected from all the animals in each group. The results were analyzed by two-way ANOVA when the effect of the treatment was investigated in time-dependent mode or by one-way ANOVA when the means of two or more samples were analyzed. All the analyses were followed by a Bonferroni post hoc test for multiple comparisons. A *p* value of less than 0.05 was considered significant. Data were analyzed using GraphPad Prism 10.6.1. (GraphPad Software, Boston, MA, USA).

## 3. Results

### 3.1. Synthesis of CAR–HNE

**Scheme 1:**
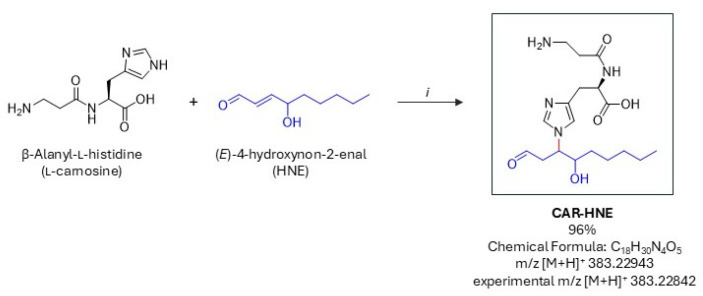
Synthesis of CAR–HNE. Reagents and conditions: (i) CH_3_CN/MeOH/H_2_O (2:1:1), room temperature, 26 h.

Nα-(3-aminopropanoyl)-Nτ-(4-hydroxy-1-oxononan-3-yl)-D-histidine (CAR–HNE) was synthesized as depicted in Scheme 1. L-carnosine was obtained from Merck, and 4-hydroxy-2(E)-nonenal (HNE) was prepared according to a previously described method [47]. HNE (700 mg, 4.48 mmol) was dissolved in 108 mL of a solvent mixture of methanol, water, and acetonitrile (1:1:2, *v*/*v*/*v*). CAR (841 mg, 3.72 mmol) was added, and the reaction mixture was stirred at room temperature for 4 h 40 min. An additional portion of HNE (147 mg, 0.941 mmol, 0.21 equivalents) was then added, and stirring was continued for a total of 26 h at room temperature. Organic solvents were removed under reduced pressure, and the remaining aqueous phase was washed with ethyl acetate (70 mL). The aqueous layer was separated and evaporated to dryness under a vacuum to afford a yellowish solid. The product showed a retention factor (Rf) of 0.404 (n-butanol/H_2_O/AcOH 3:1:1; visualized with ninhydrin) and was obtained in a 96% yield. HRMS (ESI) calculated *m*/*z* [M + H]^+^ 383.22943, experimental *m*/*z* [M + H]^+^ 383.22842.

The 1H-NMR spectrum reflects the presence of multiple species in equilibrium, likely due to tautomerism, hydration, and stereoisomerism of the CAR–HNE adduct (Figure S2, Supplementary Data).

The structural characterization of the CAR–HNE adduct has been previously comprehensively established by our group using combined ^1^H and ^13^C NMR, 2D NMR (COSY, TOCSY, HSQC, HMBC), and MS analyses [22]. Those studies demonstrated that the major product corresponds to the Michael addition of HNE to the imidazole ring of CAR, followed by intramolecular cyclization to form a 13-membered macrocyclic imine–Michael adduct. The adduct exists in equilibrium with a hemiacetal form and displays pH-dependent tautomeric behavior. In the present work, 1D NMR analysis was performed to confirm the consistency with the previously established structure, as the aim of this study was not structural re-elucidation but functional characterization.

### 3.2. Rate Constants

Kinetic analysis of the reversible reaction between CAR and HNE yielded consistent values for the association and dissociation rate constants, as well as the equilibrium dissociation constant. The second-order association rate constant kon was determined to be 0.036 M^−1^·s^−1^, based on the time-dependent depletion of HNE under pseudo-first-order conditions with CAR in molar excess. The value agrees with that already reported by Zhao, who reported a kon of 0.035 M^−1^·s^−1^ [26], and by Artasensi et al. of 0.041 M^−1^·s^−1^ [48]. The dissociation rate constant koff, calculated from the initial rate of HNE release from a pre-formed CAR–HNE adduct, was found to be 1.48 × 10^−6^ s^−1^. Using these values, the equilibrium constant was derived as 4.1 × 10^−5^ M.

These values are consistent with a low equilibrium dissociation constant and demonstrate that CAR–HNE formation is kinetically favored and thermodynamically stable.

### 3.3. Cell Toxicity

**Figure 1:**
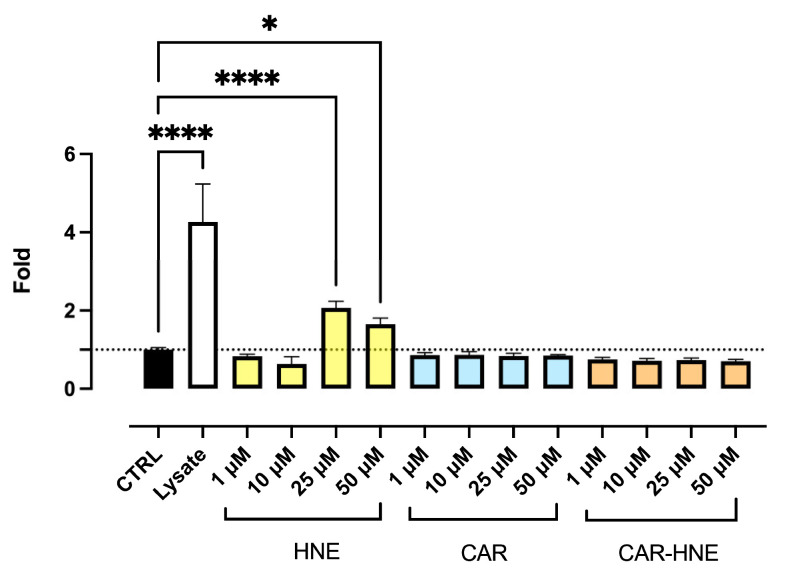
Evaluation of the cell toxicity of HNE, CAR and CAR–HNE using the CytoTox-Glo™ Cytotoxicity Assay. The assay measures dead-cell protease activity as an indicator of cell lysis/death. The white bar represents the lysate control (100% protease activity), used as the positive control to normalize the cytotoxicity response. Compounds were tested at increasing concentrations (1–50 µM). Cell toxicity was performed on HEK293T cells with Nrf2/ARE Responsive Luciferase Reporter. * *p* < 0.05; **** *p* < 0.001.

Figure 1 shows the dose-dependent cell toxic profile of CAR, HNE and CAR–HNE as determined by the CytoTox-Glo™ Cytotoxicity Assay.

As expected, CAR was found to be safe up to the highest tested concentration, according to its safe profile as already reported [49]. By contrast, HNE induced dose-dependent cell toxicity, being toxic at concentrations higher than 10 µM. It should be noted that the toxic concentration of HNE, as reported in many papers, varies on the basis of the cell type, cell density and cell toxicity assay. As the cell density increases, the cell toxicity reduces, with more metabolic cells per moles of aldehyde being able to detoxify the damaging molecule by phase I and II pathways. Moreover, the results concerning cell toxicity can greatly vary based on the cell toxicity test used. For instance, we found that the MTT assay and RealTime-Glo™ Assay, which evaluate the enzymatic reducing activity of cells, can down-estimate the cell toxicity because HNE overexpresses the reducing enzymes.

CAR–HNE showed a safe profile at least up to the highest dose tested, 50 µM, indicating that the toxic effects and reactivity of HNE are hampered when it is covalently bound to a nucleophile such as CAR.

### 3.4. Cell Studies to Assess Antioxidant and Anti-Inflammatory Activities

We then studied the effect of the three compounds as Nrf2 activators by using cell-based Nrf2/ARE reporter gene assays based on fluorescence and luminescence readouts.

**Figure 2:**
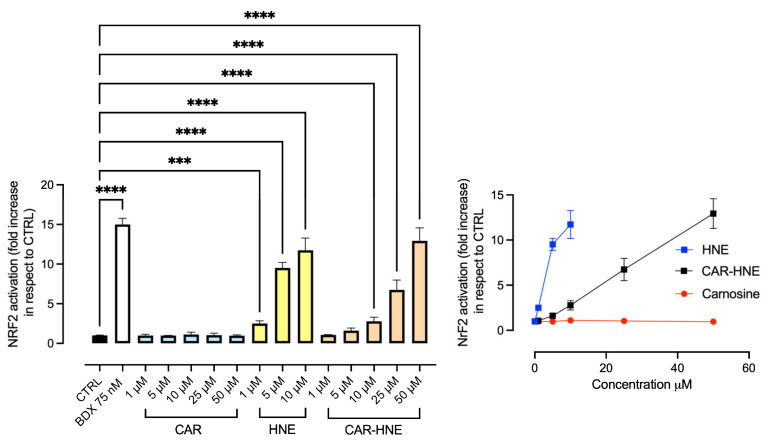
Cellular antioxidant activity. Dose-dependent antioxidant activity of CAR–HNE, CAR, and HNE in the HEK293T cell line with a gene reporter for Nrf2. Bardoxolone (BDX, 75 nM) was used as a positive control for Nrf2 activation. Results are presented as bar graphs with significance indicated ((**left**) panel) and as an XY graph ((**right**) panel) to better illustrate the dose-dependent response. Statistical significance was determined using one-way ANOVA followed by Bonferroni’s multiple comparisons test, with *p* < 0.05 considered significant. *** *p* < 0.001; **** *p* < 0.0001.

Figure 2 reports the dose-dependent Nrf2 activation. CAR was not found to be effective, demonstrating that the molecule is not a direct player in the Nrf2 activation but needs an activation process, which probably occurs in some oxidative conditions. As expected, HNE was found to be a dose-dependent activator. CAR–HNE was also found to activate Nrf2 activation, and such an effect was further confirmed using the PathHunter^®^ Keap1–Nrf2 nuclear translocation assay, which specifically reports on disruption of the Keap1–Nrf2 interaction (Figure S3, Supplementary Data). While this assay supports engagement of the Keap1-dependent regulatory axis, it does not provide direct molecular evidence of KEAP1 cysteine alkylation.

The ability of CAR–HNE to activate Nrf2 may be mechanistically related to its slow and time-dependent release of HNE via a retro-Michael reaction. The released electrophile could, in principle, interact with reactive cysteine residues within KEAP1, thereby contributing to Nrf2 stabilization. However, direct evidence of KEAP1 cysteine modification was not assessed in the present study.

**Figure 3:**
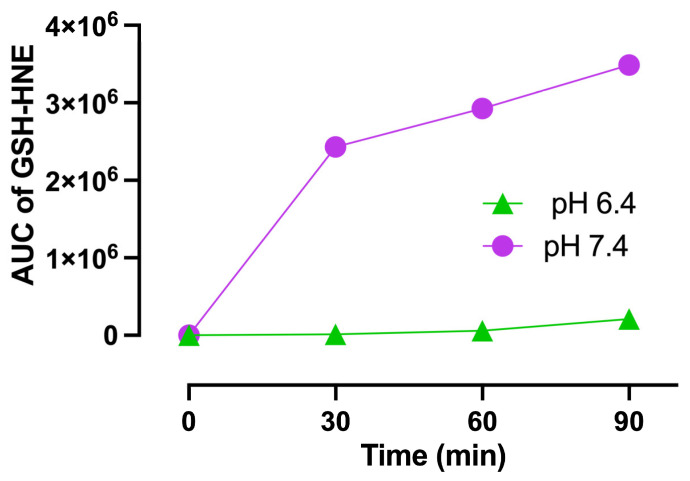
HNE transfer from CAR–HNE to GSH. Time-dependent formation of GSH–HNE by incubating CAR–HNE with GSH incubated at pH 6.4 (triangle) and 7.4 (circle). GSH–HNE was detected by monitoring the ion current of the molecular ion at *m*/*z* 464 by LC-ESI-MS using an Orbitrap as the mass analyzer. Data are reported as AUC of the peak relative to GSH–HNE.

The HNE transfer from CAR–HNE to a thiol was then studied by using GSH as an acceptor and by monitoring, by HPLC-ESI-MS analysis, the linear and time-dependent formation of the GSH–HNE adduct when incubating CAR–HNE with GSH. The formation of the GSH–HNE adducts is then due to the HNE release by CAR–HNE followed by its covalent adduction with the thiolate of GSH. The rate of GSH–HNE formation was found to be related to the pH of the medium, with the rate at pH 7.4 being significantly increased in respect to that determined at pH 6.4 (Figure 3).

Such an increase is explained by considering that according to the pKa of the thiol of GSH (pKa = 8.83), the increase of the pH from 6.4 to 7.4 increases the relative amount of the thiolate form (the reactive nucleophile able to covalently react with HNE) from 0.37 to 3.6%. Hence, the data indicate that the rate of HNE transfer of HNE from CAR–HNE is regulated by the acidity of the target thiol. The peculiarly lower pKa values reported for certain KEAP1 cysteines (e.g., C273, C288, C297, and C613), due to their proximity to basic residues, suggest that these thiols could represent preferential targets for electrophile transfer [50]. However, specific modification of KEAP1 by CAR–HNE-derived HNE was not directly measured in this study.

**Figure 4:**
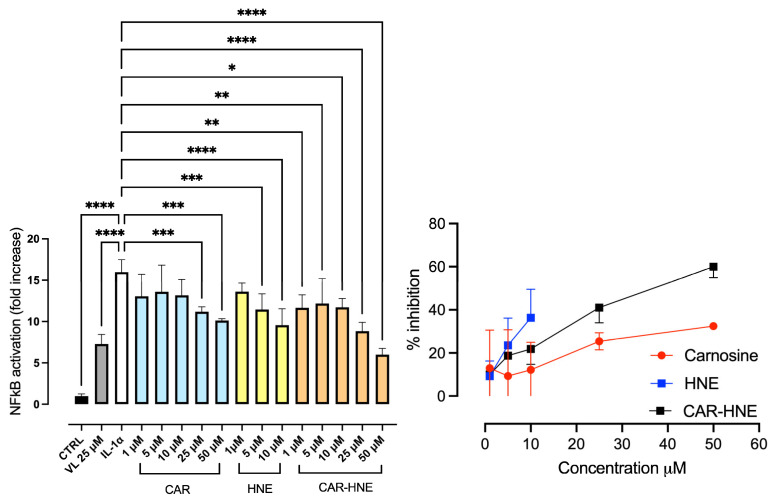
Cell anti-inflammatory activity. Dose-dependent effect of CAR–HNE, CAR, and HNE on NF-κB activation in HEK293T induced by IL-1α. 5-(3′,4′-dihydroxyphenyl)-γ-valerolactone (VL, 25 µM) was used as a positive control for its anti-inflammatory activity mediated through Nrf2 activation, as previously reported. Results are presented as bar graphs with significance indicated (left panel) and as an XY graph (right panel) to better illustrate the dose-dependent response. Statistical significance was determined using one-way ANOVA followed by Bonferroni’s multiple comparisons test, with *p* < 0.05 considered significant. * *p* < 0.05; ** *p* < 0.01; *** *p* < 0.001; **** *p* < 0.0001.

In the next step, we evaluated the effect of CAR, HNE and CAR–HNE on the Nf-kB activation as stimulated by IL-1α (Figure 4). IL-1α induced a significant NF-κB nuclear translocation, which increased 15 fold in respect to control. 5-(3′,4′-dihydroxyphenyl)-γ-valerolactone (VL) was used as the standard and reduced by almost 52% the NF-κB translocation at a concentration of 25 µM, as already reported [40]. The dose-dependent inhibitory effect of CAR, CAR–HNE and HNE on NF-κB activation is reported as a bar graph (left panel) as well as an XY chart (right panel).

It is evident that up to 10 µM, HNE is the most potent, but higher concentrations were not tested due to the toxicity. CAR–HNE was found to be effective already at 1 µM to increase the activity dose-dependently up to the highest tested dose, 50 µM. CAR was also found to be effective even with a lower potency in respect to CAR–HNE, being effective from 25 µM.

### 3.5. Metabolic Stability of CAR–HNE and Bioavailability Studies

**Figure 5:**
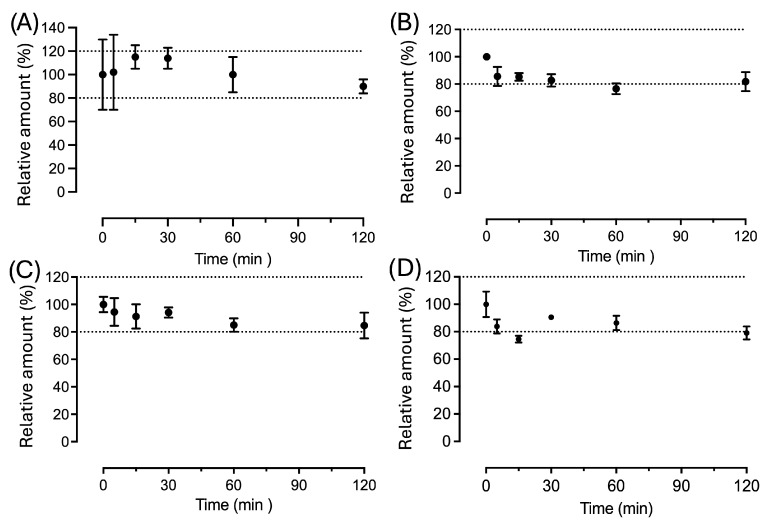
CAR–HNE metabolic stability. Time-dependent stability of CAR–HNE in different biological matrices. CAR–HNE was spiked to the biological matrices at a final concentration of 5 µM and the relative content determined by LC-ESI-MS/MS, as reported in the method session. (**A**) Human serum, (**B**) rat liver, (**C**) rat kidney, and (**D**) rat heart.

Due to their reactivity, RCS readily bind to nucleophilic substrates in tissues, resulting in a very short half-life and limited diffusion. This is evident due to the short half-life of HNE in serum and blood, as previously reported [51]. We found that CAR–HNE complexes are considerably stable in various biological matrices, including plasma, liver homogenate, and heart tissue, as the residual amount in the different matrices is almost 80% of the initial dose after 120 min of incubation (Figure 5).

The observed stability indicates that, once formed, CAR–HNE persists in biological matrices despite the presence of abundant nucleophiles. Notably, plasma represents a highly thiol-rich environment due to the abundance of albumin, whose Cys34 residue constitutes a major circulating nucleophilic sink. Despite this strong nucleophilic background, CAR–HNE exhibits measurable persistence, indicating that rapid retro-Michael dissociation followed by immediate thiol trapping is not a dominant process under the tested conditions. These findings support the relative stability of the adduct while remaining consistent with a slow electrophile-transfer mechanism responsible for modulating a nucleophilic tone by activating Nrf2.

### 3.6. In Vivo Studies

#### 3.6.1. Effect of HNE, CAR and CAR–HNE Administration on Macroscopic and Microscopic Alteration, Neutrophil Infiltration, Animal Body Weight Increase and Colon Length in DSS Mice

**Figure 6:**
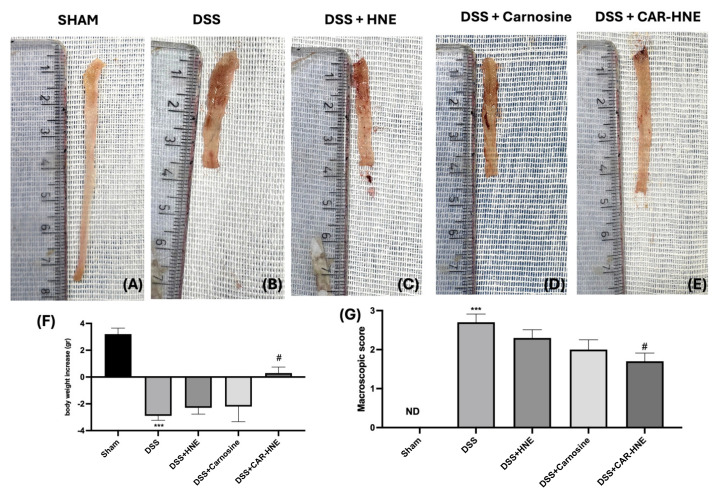
Effects of HNE, CAR and CAR–HNE treatments on macroscopic damage score and body weight gain. Colon tissues from sham-treated mice (**A**), colon tissues from DSS-treated mice (**B**) and colon tissues collected from DSS-treated mice that received HNE at a dose of 5.77 mg/kg (**C**), CAR at a dose of 8.36 mg/kg (**D**) and CAR–HNE at a dose of 14.5 mg/kg (**E**). Macroscopic damage score (**G**) was performed by two independent observers. Body weight gain (**F**). Data are mean ± SEM of 6 mice in each group. *** *p* < 0.001 vs. sham # *p* < 0.05 vs. DSS. ND: not detectable.

The colon of the sham group showed no macroscopic alterations (Figure 6A, see macroscopic score Figure 6G). In contrast, following administration of DSS, the colons appeared with inflammation and hyperemia (Figure 6B). Oral administration of HNE and CAR at doses of 5.77 mg/kg and 8.36 mg/kg showed a slight reduction in inflammation and macroscopic alteration, while CAR–HNE at a dose of 14.5 mg/kg significantly reduced macroscopic alteration in DSS-treated mice compared to the sham group (Figure 6A–E, see macroscopic score Figure 6G).

**Figure 7:**
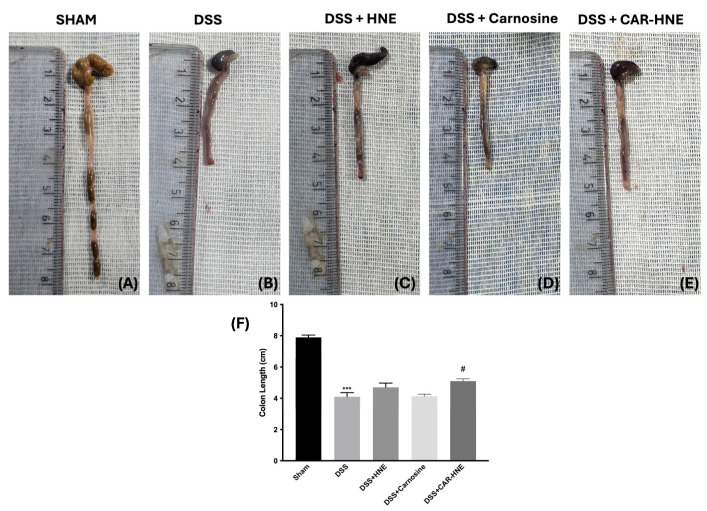
Effects of HNE, CAR and CAR–HNE treatments on colon length. Colon tissues from sham-treated mice (**A**), colon tissues from DSS-treated mice (**B**) and colon tissues collected from DSS-treated mice that received HNE at a dose of 5.77 mg/kg (**C**), CAR at a dose of 8.36 mg/kg (**D**) and CAR–HNE at a dose of 14.5 mg/kg (**E**). Colon length score (**F**). Data are mean ± SEM of 6 mice in each group. *** *p* < 0.001 vs. sham # *p* < 0.05 vs. DSS.

Furthermore, all the DSS-treated mice experienced weight loss compared to the sham group, although oral CAR–HNE significantly attenuated this loss (Figure 6F). Overall, CAR–HNE alleviated the clinical signs of colonic inflammation (Figure 6). In addition, DSS-treated mice presented reduced colon length while CAR–HNE-treated mice significantly showed a greater length of the colon compared to the other groups (Figure 7A–E and see graph Figure 7F).

**Figure 8:**
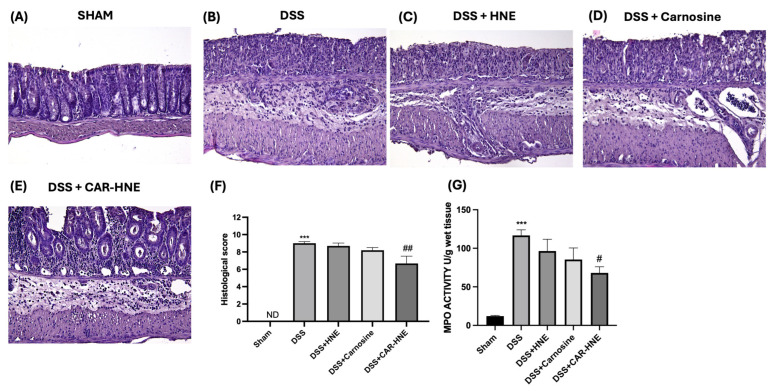
Effects of HNE, CAR and CAR–HNE treatments on colonic lesions, histological score and neutrophil infiltration. Histological analysis performed in the colon section from sham-treated mice (**A**), from DSS-treated mice (**B**) and from DSS-treated mice with HNE at a dose of 5.77 mg/kg (**C**), with CAR at a dose of 8.36 mg/kg (**D**) and with CAR–HNE at a dose of 14.5 mg/kg (**E**). The histological score (**F**) was performed by two independent observers. MPO assay measurement (**G**). Figures are representative of three independent staining performed on different days. Magnification 20×, scale bar 100 µm. Data are mean ± SEM of 6 mice in each group. *** *p* < 0.001 vs. sham, ## *p* < 0.01 vs. DSS. *** *p* < 0.001 vs. sham, # *p* < 0.05 vs. DSS. ND: not detectable.

Histological analysis showed no structural changes in the colonic tissue of sham mice (Figure 8A and corresponding histological score, Figure 8F). In contrast, DSS-treated mice showed epithelial damage, destruction of the colon architecture and leucocyte infiltration (Figure 8B and corresponding histological score, Figure 8F). Oral administration of HNE and CAR slightly reduced these histological changes, whereas administration of CAR–HNE significantly reduced colon inflammation, oedema and leucocyte infiltration (Figure 8C–F). The colitis caused by DSS was also characterized by an increase in myeloperoxidase activity, an indicator of the neutrophil accumulation in the colon. According to the MPO assay, neutrophil infiltration was much lower in mice treated with CAR–HNE than in mice treated with DSS (Figure 8G).

#### 3.6.2. Proteomic Studies

The here reported proteomic approach provided comprehensive molecular insights, allowing us to characterize the effects of CAR–HNE on the colonic proteome. On one hand, the data support the protective role of CAR–HNE in reducing the inflammatory and damaging effects of DSS; on the other, they corroborate its antioxidant and anti-inflammatory mechanism of action, which appears to involve activation of the Nrf2 pathway.

**Figure 9:**
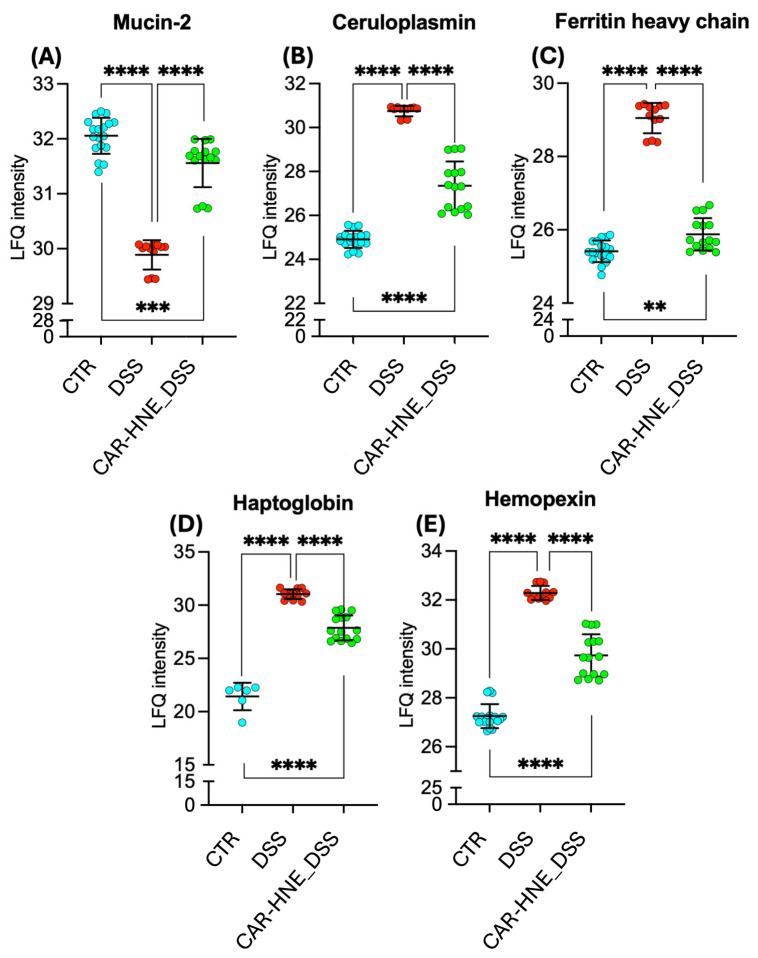
CAR–HNE treatment modulates the expression of proteins associated with mucosal integrity, inflammation, and iron metabolism in a DSS-induced colitis model. (**A**) The mucin-2 levels, significantly reduced by DSS, were partially restored by CAR–HNE. (**B**) Ceruloplasmin and (**C**) ferritin heavy chain were upregulated in the colitic mice and decreased upon treatment. Similarly, the DSS-induced increase in (**D**) haptoglobin and (**E**) hemopexin was significantly attenuated by CAR–HNE. Data are shown as LFQ intensities (median with interquartile range). ** *p* < 0.01; *** *p* < 0.001; **** *p*  <  0.0001.

In the DSS-induced colitis model, one of the most significant findings was the marked downregulation of Muc2, a key protein responsible for mucus secretion (Figure 9, panel A). This reduction was associated with impaired intestinal barrier integrity and increased epithelial permeability. Treatment with CAR–HNE restored Muc2 expression, indicating a potential improvement in mucosal barrier function (Figure 9, panel A).

The compromised intestinal barrier function in the DSS model was associated with the molecular signatures of both ferroptosis and heme-driven toxicity. Proteomic analysis revealed elevated levels of ceruloplasmin and ferritin heavy chain 1 (Fth1), two key proteins involved in iron homeostasis and oxidative stress, indicating intracellular iron accumulation and lipid peroxidation typical of ferroptotic processes (Figure 9, panel B,C). In parallel, the increased expression of hemopexin and haptoglobin—extracellular scavengers of free heme and hemoglobin—reflected a systemic response to heme overload and its pro-oxidant effects (Figure 9, panel D,E). Treatment with CAR–HNE markedly downregulated all four proteins, suggesting a coordinated reduction of iron and heme toxicity (Figure 9, panel B–E). These changes point to improved control of redox homeostasis and significant attenuation of oxidative and inflammatory damage in the colonic microenvironment.

**Figure 10:**
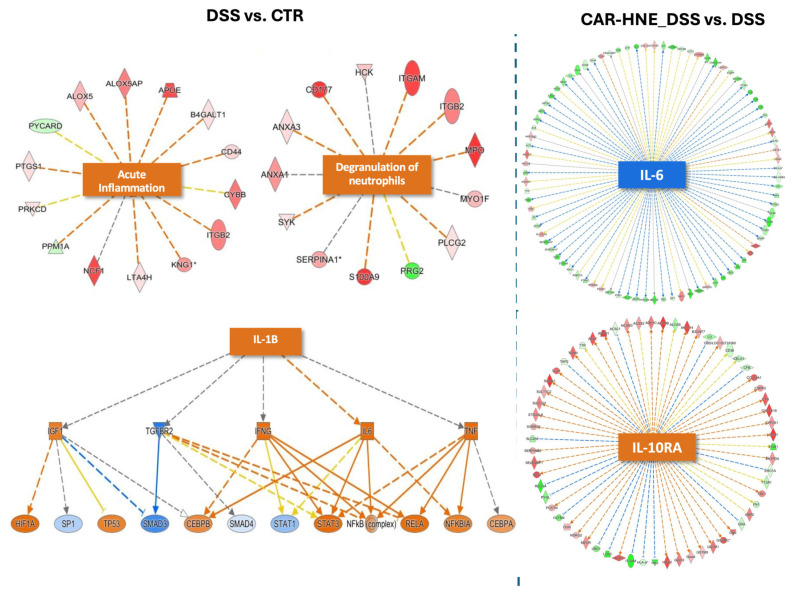
CAR–HNE modulates inflammatory pathways in DSS-induced colitis as revealed by Ingenuity Pathway Analysis (IPA). IPA^®^ was used to identify inflammatory mechanisms triggered by DSS and modulated by CAR–HNE treatment. (**Left**) panels (DSS vs. CTR): DSS exposure was associated with activation of acute inflammatory responses, neutrophil degranulation, and IL-1β-related signaling, consistent with the increased abundance of pro-inflammatory proteins (red nodes) and predicted pathway activation (orange, predicted activation). (**Right**) panels (DSS + CAR–HNE vs. DSS): CAR–HNE attenuated inflammatory signaling, with inhibition of the IL-6 axis (blue, predicted inhibition) and activation of IL-10RA (orange, predicted activation), supporting a shift toward mucosal immune tolerance. Color code: node fill indicates measured expression changes (red, increased; green, decreased; color intensity reflects magnitude). Predicted functional states are shown as orange (activation) and blue (inhibition; color intensity reflects prediction confidence). Edges are coded as orange lines (leads to activation), blue lines (leads to inhibition), yellow lines (findings inconsistent with the downstream state), and gray lines (effect not predicted). The asterisk next to a molecule name indicates that multiple input identifiers map to the same entity in the IPA knowledge base (i.e., a duplicate mapping). Analyses were performed using a threshold of *p* < 0.05 and activation z-score ≥ |2|.

Ingenuity Pathway Analysis (IPA^®^) revealed modulation of heme-associated responses across experimental groups. In DSS-treated animals, IPA^®^ predicted activation of the “phagocytosis of red blood cells” pathway, suggesting increased hemolytic stress and tissue damage. Furthermore, IPA^®^ software showed that inflammation in the DSS-induced colitis model was characterized by marked immune cell extravasation, activation of the neutrophil degranulation pathway, and elevated levels of pro-inflammatory mediators, including IL-1β (Figure 10, left panel). As illustrated in the IPA-derived wheel-shaped network, treatment with CAR–HNE suppressed heme-related signaling networks, including the Hbb-b2-centered interaction hub and effectively attenuated this acute inflammatory response and reduced IL-6 signaling, suggesting a mitigation of sustained inflammatory damage (Figure S4, Supplementary Data). Furthermore, the anti-inflammatory effect of CAR–HNE was supported by the upregulation of the IL-10Rα pathway, a key regulator of intestinal immune tolerance and mucosal homeostasis (Figure 10, right panel). These findings point to reduced heme release and enhanced control of heme-driven oxidative stress.

Proteomic analysis and Ingenuity Pathway Analysis also revealed modulation of wound healing and coagulation-associated responses.

**Figure 11:**
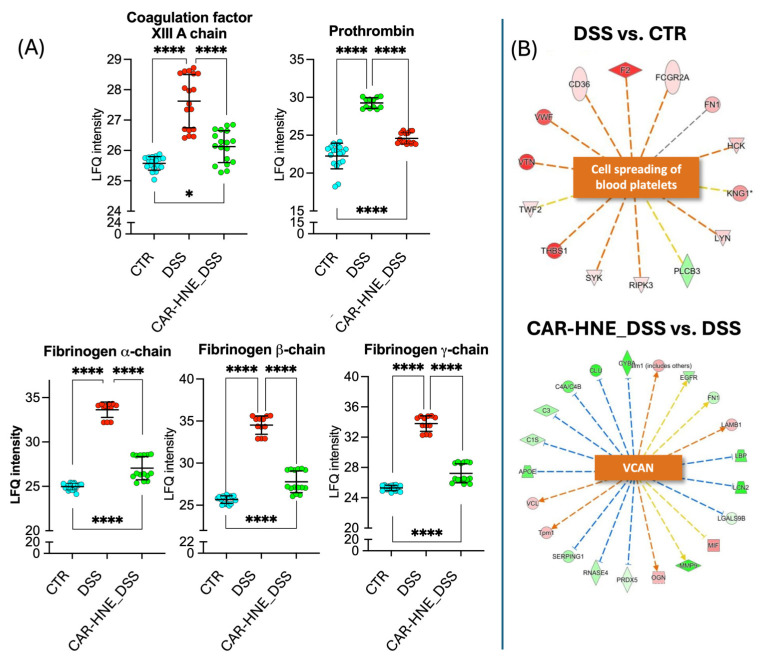
CAR–HNE reduces activation of coagulation and tissue repair pathways in DSS-induced colitis. Proteomic analysis and Ingenuity Pathway Analysis (IPA^®^) revealed modulation of wound healing and coagulation-associated responses. Panel (**A**): DSS treatment induced a strong upregulation of coagulation factors (e.g., prothrombin [F2], fibrinogen chains [FGA, FGB, FGG]) and extracellular matrix proteins (e.g., vitronectin [VTN], fibronectin [FN1]), indicating active tissue damage and remodeling. CAR–HNE significantly reduced the expression of all the upregulated proteins. Panel (**B**) (IPA^®^): Pathway enrichment analysis predicted activation of platelet spreading and wound repair processes in DSS vs. control (**A**), while CAR–HNE treatment reversed this pattern, downregulating repair-associated proteins, including versican (VCAN) and several ECM remodeling components (**B**). Node colors indicate protein abundance (red: upregulated; green: downregulated), and edge colors reflect predicted functional states (orange: activation; blue: inhibition). LFQ intensity values are displayed as box plots. * *p* < 0.05; **** *p* < 0.0001.

DSS treatment induced a strong upregulation of coagulation factors (e.g., prothrombin [F2], fibrinogen chains [FGA, FGB, FGG]) and extracellular matrix proteins (e.g., vitronectin [VTN], fibronectin [FN1]), indicating active tissue damage and remodeling (Figure 11).

Pathway enrichment analysis predicted activation of platelet spreading and wound repair processes in DSS vs. control, while CAR–HNE treatment reversed this pattern, downregulating repair-associated proteins, including versican (VCAN) and several ECM remodeling components (Figure 11).

**Figure 12:**
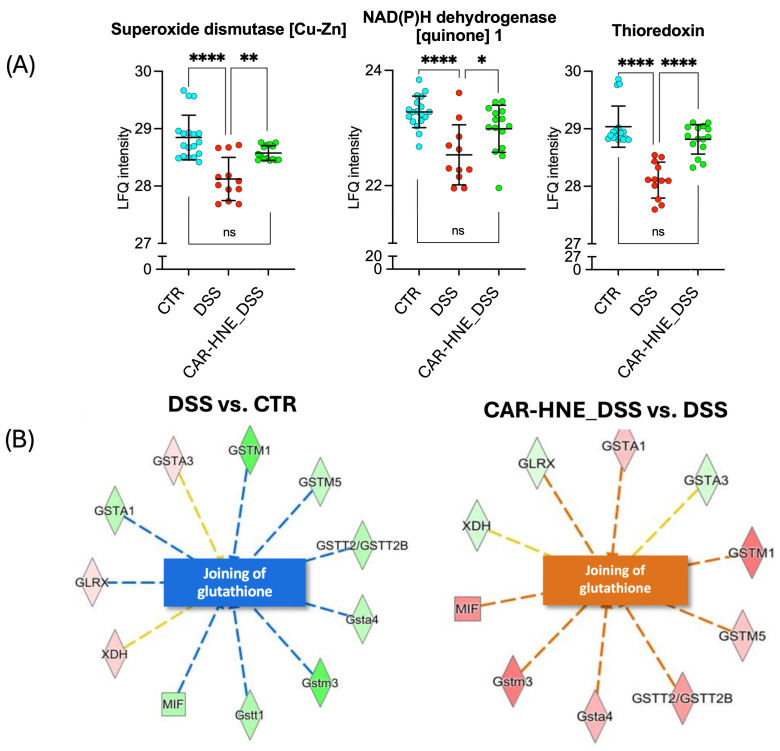
CAR–HNE activates the Keap1–Nrf2 antioxidant pathway and restores redox homeostasis. Proteomic and Ingenuity Pathway Analysis (IPA^®^) of colonic tissue revealed modulation of key antioxidant mechanisms following DSS and CAR-HNE treatment. Panel (**A**): DSS exposure led to significant downregulation of several Nrf2-dependent antioxidant enzymes, including glutathione S-transferase isoforms (GSTs), superoxide dismutase 1 (SOD1), thioredoxin-1 (TXN1), and NAD(P)H quinone dehydrogenase 1 (NQO1), indicating impaired oxidative stress responses. Panel (**B**) (IPA^®^): In the DSS vs. CTR comparison, antioxidant activity and the “joining of glutathione” pathway were predicted to be inhibited (**left**). Treatment with CAR–HNE reversed this pattern, activating the Keap1–Nrf2 pathway and restoring expression of antioxidant enzymes involved in glutathione metabolism (**right**). Node color indicates differential protein abundance (red: upregulated; green: downregulated), while edge color reflects predicted activity states (orange: activation; blue: inhibition). Box plots show LFQ intensities for representative proteins. * *p* < 0.05; ** *p* < 0.01; **** *p* < 0.0001.

CAR–HNE treatment appeared to exert upstream regulation by activating the Nrf2–Keap1 signaling axis, a master regulator of the cellular antioxidant response. This activation was associated with increased expression of several Nrf2-dependent antioxidant genes, including multiple isoforms of glutathione S-transferases (GSTs), which contribute to the detoxification of reactive electrophiles and peroxides. Notably, the restoration of superoxide dismutase 1 (SOD1), thioredoxin-1 (TXN1), and NAD(P)H quinone dehydrogenase 1 (NQO1) further confirmed reactivation of the redox defense system (Figure 12).

Together, these changes indicate that CAR–HNE enhances endogenous antioxidant capacity, thereby mitigating oxidative damage and improving cellular resilience in inflamed colonic tissue.

## 4. Discussion

CAR is a somewhat enigmatic endogenous molecule. To date, a substantial body of evidence suggests that it acts as an antioxidant and anti-inflammatory agent, though the mechanisms through which it exerts these effects remain unclear [1].

In this paper, we propose that the anti-inflammatory and antioxidant activity of CAR appears to be triggered under oxidative stress conditions.

In particular, the action of CAR starts with its reaction with reactive carbonyl species (RCS), particularly α,β-unsaturated aldehydes, which are formed as lipid-peroxidation by-products. The occurrence of such reactions in vivo is proven by the detection of CAR adducts with HNE and acrolein in both animal and human studies [17,18,19,20,52]. These findings, consistently reproduced across independent studies, establish that CAR–HNE formation is a biologically occurring event under conditions of oxidative stress and not merely a chemical possibility observed in vitro. Based on the RCS-sequestering ability of CAR, some of its protective antioxidant effects have been attributed to its ability to quench RCS, as proposed in several of our previous studies. However, considering the reaction rate constants of RCS with CAR compared with thiol substrates, the quenching effect alone is unlikely to be quantitatively sufficient to fully account for its biological effects. Instead, we propose that CAR reacts with a (minor) fraction of RCS, generating Michael adducts whose biological activity, rather than their mere formation, represents the focus of the present investigation.

Once formed, the CAR–RCS adducts exist in reversible equilibrium with a small fraction of free aldehyde. In the presence of reactive thiolates, electrophile transfer may occur according to kinetic and thermodynamic principles. This mechanism is consistent with an electrophile-mediated activation of the Keap1–Nrf2 pathway, although direct modification of specific KEAP1 cysteine residues was not experimentally demonstrated in the present work. This process maintains nucleophilic tone through Nrf2 activation, which in turn modulates NF-κB activity, ultimately reducing inflammation and improving antioxidant defenses.

It is known that free RCS also activate Nrf2; however, their effect is limited by their hormetic response and metabolic instability. In contrast, CAR–HNE does not exhibit a damaging response and is relatively stable, as evidenced by the following findings:

1. The hormetic effect of HNE was demonstrated by its ability to induce an antioxidant (Nrf2 activation) and anti-inflammatory response at concentrations up to 10 μM, whereas it became cytotoxic at higher concentrations—an effect already reported in numerous studies.
2. CAR–HNE, like HNE, was protective at concentrations up to 10 μM and even at higher concentrations but was not found to be toxic, at least up to 50 μM.

Taken together, these data demonstrate that the covalent CAR–HNE adduct itself is not reactive or cytotoxic. Instead, its antioxidant and anti-inflammatory action appears consistent with a slow and regulated electrophile-transfer process, maintaining the nucleophilic tone without reaching toxic levels of free aldehydes. Consequently, the reaction of CAR with RCS shifts the hormetic effect of RCS, as observed in various models, toward a protective outcome without the associated damaging effects.

A crucial aspect to consider in the protective mechanism of CAR–HNE is the rate of HNE release, which is driven by the stability of the complex and the pKa of the SH group of the target of HNE. The dissociation rate constant koff was found to be 1 × 10^−6^ s^−1^ [20,40], indicating a low equilibrium dissociation constant and demonstrating that CAR–HNE formation is kinetically favored and thermodynamically stable. After 2 h of CAR–HNE incubation, only 1.07% of the adduct had dissociated, corresponding to approximately 2 μM of HNE, while at 24 h, the dissociation reached 7.62%. Once released, HNE reacts with nucleophiles such as cysteine residues. The reaction kinetics are governed by the concentration of the thiolate anion (S^−^), the reactive nucleophilic species, which depends not only on the total thiol concentration but also on its acidity (pKa). This mechanism was well described using glutathione (GSH) as a thiol nucleophilic model, measuring the rate of GSH–HNE adduct formation via mass spectrometry (MS) upon incubation of CAR–HNE with GSH at different pH values (6.4 and 7.4). The pKa of GSH is 8.83, and at pH 7.4, according to the Henderson–Hasselbalch equation, there are 3.7 moles of GS^−^ per 100 moles of GSH. By reducing the pH to 6.4, the amount of GS^−^ reduces by almost 100 fold, being 0.37 moles per 100 moles of GSH. The thiolate/thiol ratio parallels the rate of GSH–HNE formation, with this last being almost 100 fold higher at pH 7.4 in respect to 6.4, demonstrating that thiol acidity drives HNE release. These data suggest that CAR–HNE transfers HNE preferentially toward the most reactive (acidic) thiols.

Another important aspect to consider is the metabolic stability of CAR–HNE, as opposed to free HNE, which is rapidly metabolized by phase I and II reactions. The enhanced stability of CAR–HNE compared to free HNE can be rationalized by straightforward structural considerations. HNE is an α,β-unsaturated aldehyde and therefore a potent Michael acceptor, characterized by a highly electrophilic β-carbon. Upon conjugation with CAR, the α,β-unsaturated double bond is consumed, eliminating the primary electrophilic center responsible for the high reactivity of free HNE. Therefore, the adduct no longer retains the strong intrinsic electrophilic character of HNE, accounting for its increased persistence and reduced nonspecific reactivity in biological matrices.

The metabolic stability of CAR–HNE, as demonstrated in various biological matrices, allows the nucleophilic tone to extend over inflammatory foci, thereby preventing the spread of inflammation and oxidative stress. Notably, CAR–HNE is stable in human serum, indicating that when HNE is bound to CAR, it is not recognized by carnosinases, which selectively cleave the peptidic bond of CAR. Furthermore, considering the stability of CAR–HNE in human serum, as well as in liver—a tissue rich in glutathione (GSH)—the measurable persistence of the adduct, despite the abundance of nucleophilic thiols such as albumin Cys34 in plasma, suggests that electrophile transfer to endogenous thiols is kinetically limited under these conditions.

The detection of CAR–HNE in urine in previous studies [19,20] further indicates that the adduct can enter the systemic circulation and be transported beyond its site of formation. While this does not establish targeted distal signaling, it supports the notion that CAR–HNE is sufficiently stable to persist and undergo systemic redistribution in vivo.

The antioxidant and anti-inflammatory action of CAR–HNE was tested in cell-based models. In the cellular model used in this study, which was not subjected to oxidative stress or lipid peroxidation, endogenous HNE formation is expected to be minimal. Under these basal conditions, CAR alone, being a nucleophilic peptide lacking intrinsic electrophilic properties, would not be expected to activate Nrf2 through direct KEAP1 cysteine modification. In contrast, CAR–HNE, which retains electrophilic character, activates Nrf2 in a manner consistent with electrophile-mediated signaling. These findings support the concept that CAR-dependent Nrf2 activation is redox-context dependent.

The antioxidant properties of CAR–HNE contributed to the anti-inflammatory effect, as evidenced by its ability to suppress NF-κB activity in cells equipped with an NF-κB reporter system and challenged with IL-1α. Interestingly, CAR was also found to be effective as an anti-inflammatory agent, although with much lower potency compared to CAR–HNE. The anti-inflammatory effect of CAR may be mediated by its in situ conversion, within inflamed cells exposed to the inflammatory cascade, into CAR–HNE or other related adducts as a consequence of HNE/RCS generation by the inflammation-induced oxidative stress.

A well-established interplay exists between oxidative stress and inflammation, as well as between the two key transcription factors involved in these processes—NF-κB and Nrf2. When oxidative stress occurs as a primary disturbance, it often leads to secondary inflammation, which in turn exacerbates oxidative stress. Conversely, when inflammation is the primary disorder, it can trigger oxidative stress as a secondary response, further amplifying the inflammatory state.

The antioxidant and anti-inflammatory activity of CAR–HNE was then tested in vivo in a model of colon inflammation. Based on our previous pharmacokinetic observations indicating limited systemic absorption of exogenous CAR–HNE, DSS-induced colitis was selected as a proof-of-concept model to assess local biological activity in the gastrointestinal compartment under inflammatory stress. Colon damage, as determined by macroscopic score, was found to be reduced by CAR–HNE treatment but not by an equal stoichiometric amount of CAR and HNE, indicating a synergistic action when covalently bound. Furthermore, the inflammatory response induced by DSS was significantly reduced by CAR–HNE but not by the two reactants separately. A limitation of this study is that CAR–HNE levels were not quantified in plasma or colon tissue after dosing; therefore, the tissue exposure and half-life remain to be determined.

The molecular pathways regulated by CAR–HNE were then elucidated using a proteomic approach, demonstrating its anti-inflammatory efficacy through Nrf2 pathway activation and the restoration of intestinal homeostasis.

The mechanism discussed here may help interpret previous in vivo studies reporting the anti-inflammatory and antioxidant actions of CAR, linking the already demonstrated formation of CAR–HNE to its biological consequences.

A key strength of the present study lies in the application of an unbiased quantitative proteomic approach to characterize the in vivo molecular effects of CAR–HNE in inflamed colonic tissue. Unlike targeted biochemical assays focused on single endpoints, the nano-LC-HRMS workflow combined with LFQ-based analysis allowed the simultaneous assessment of coordinated protein networks involved in redox homeostasis, iron metabolism, inflammation, coagulation, and tissue remodeling. Importantly, the proteomic signature observed in DSS-treated animals was consistent with a redox-imbalanced and pro-inflammatory state, characterized by suppression of canonical antioxidant enzymes and activation of acute phase, heme-scavenging, and wound-healing pathways. CAR–HNE treatment produced a coherent and system-level reprogramming of these networks, restoring Nrf2-dependent antioxidant proteins (e.g., GST isoforms, SOD1, TXN1, NQO1) while attenuating IL-6-driven inflammatory signaling and coagulation-associated responses.

Notably, the convergence between the functional assays (Nrf2 reporter activation, NF-κB inhibition) and proteomic pathway analysis strengthens the mechanistic interpretation that CAR–HNE acts upstream at the level of redox-sensitive signaling, rather than merely exerting a downstream anti-inflammatory effect. The coordinated modulation of iron-handling proteins (ceruloplasmin, ferritin heavy chain), heme scavengers (haptoglobin, hemopexin), and antioxidant enzymes further supports the concept that CAR–HNE improves the “nucleophilic tone” of the tissue microenvironment, consistent with a controlled electrophile-transfer mechanism.

Although the proteomic analysis was performed on pooled experimental groups and requires further validation at the individual protein level, the system-level consistency of the regulated pathways provides robust molecular support for the proposed redox-tunable signaling model of CAR–HNE.

At present, quantitative data on endogenous CAR–HNE concentrations in vivo remain limited, and the physiological levels under different pathological conditions are not yet fully defined. Nevertheless, the reproducible detection of CAR–HNE in biological matrices supports its formation under oxidative stress. In this context, the present findings can be interpreted with a dual perspective: on one hand, they suggest that CAR–HNE may act as an endogenous redox mediator contributing to CAR-dependent cytoprotection; on the other, they open up the possibility of considering CAR–HNE and structurally related Michael adducts as a novel class of redox-tunable Nrf2 activators with potential pharmacological relevance. This concept warrants further medicinal chemistry investigation, which is currently underway in our laboratory.

### Limitations

There is inter-animal variation in the severity of the disease in the DSS-induced colitis model, which could lead to variation in the outcomes that are evaluated. Furthermore, this model might not accurately represent the long-term pathological characteristics of human ulcerative colitis because it mainly simulates acute intestinal inflammation. Although CAR–HNE showed high stability in biological matrices, the present work did not include a full pharmacokinetic/biodistribution characterization in vivo (e.g., systemic exposure, tissue distribution, and clearance), nor a comprehensive evaluation of potential downstream metabolites.

## Data Availability

The mass spectrometry proteomics data have been deposited into the ProteomeXchange Consortium via the PRIDE [53] partner repository with the dataset identifier PXD075159.

## Supplementary Materials

The following supporting information can be downloaded at https://www.mdpi.com/article/10.3390/antiox15030388/s1, Figure S1: Animal treatment scheme; Figure S2: ^1^H NMR spectrum of CAR–HNE; Figure S3: Dose-dependent activation of Nfr2 induced by CAR–HNE and HNE as determined by the PathHunter Enzyme Fragment Complementation Assay Platform; Figure S4: Treatment with CAR–HNE suppressed heme-related signaling networks, including the Hbb-b2-centered interaction hub, suggesting a mitigation of sustained inflammatory damage.

## Abbreviations

The following abbreviations are used in this manuscript:

CAR	carnosine
CAR-HNE	Michael adduct of carnosine with 4-hydroxy-2-nonenal
DSS	dextran sodium sulfate
HNE	4-hydroxy-2-nonenal
RCS	reactive carbonyl species
