# Supplementary material for: The Carnosine–HNE Michael Adduct as a Redox-Active Species Associated with Nrf2-Dependent Antioxidant and Anti-Inflammatory Responses
Source: Antioxidants (Basel). 2026 Mar 19;15(3):388. doi: 10.3390/antiox15030388 (PMC13024699; doi:10.3390/antiox15030388)
Supplement: Supplementary file 1 [file antioxidants-15-00388-s001.zip › antioxidants-4155293-supplementary.pdf]

Supplementary Data

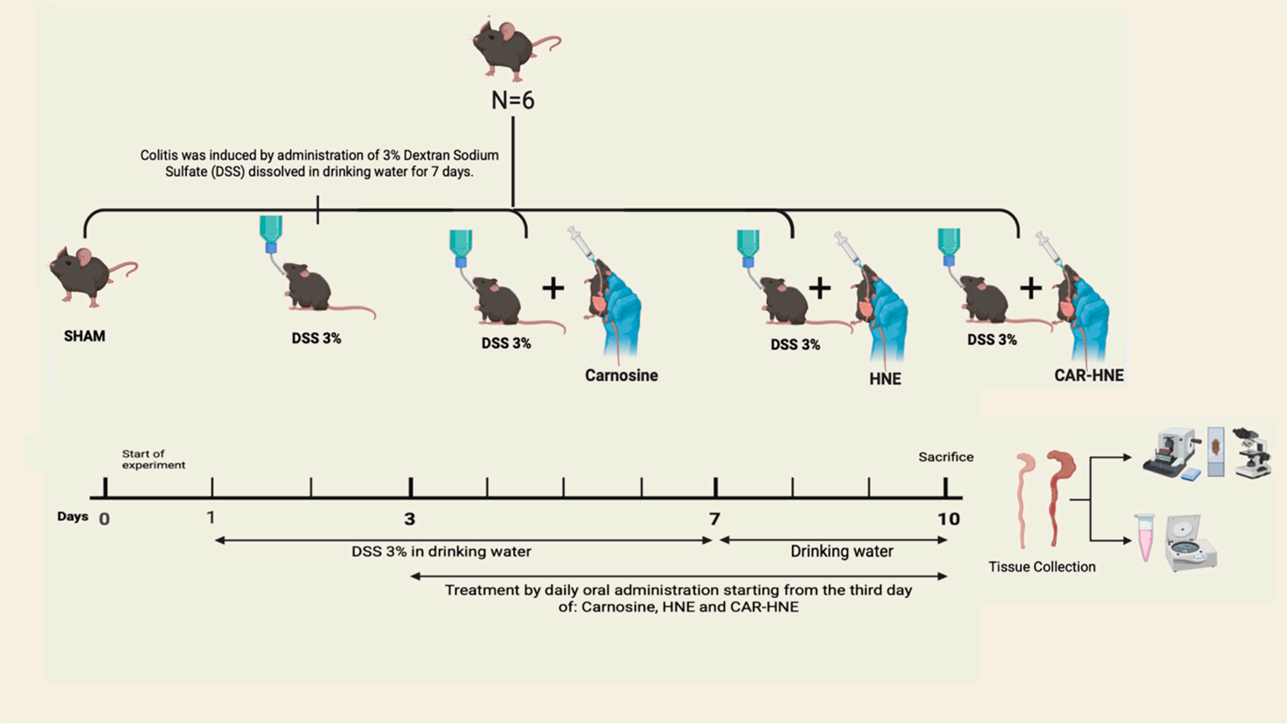

Figure S1 – Animal treatment scheme

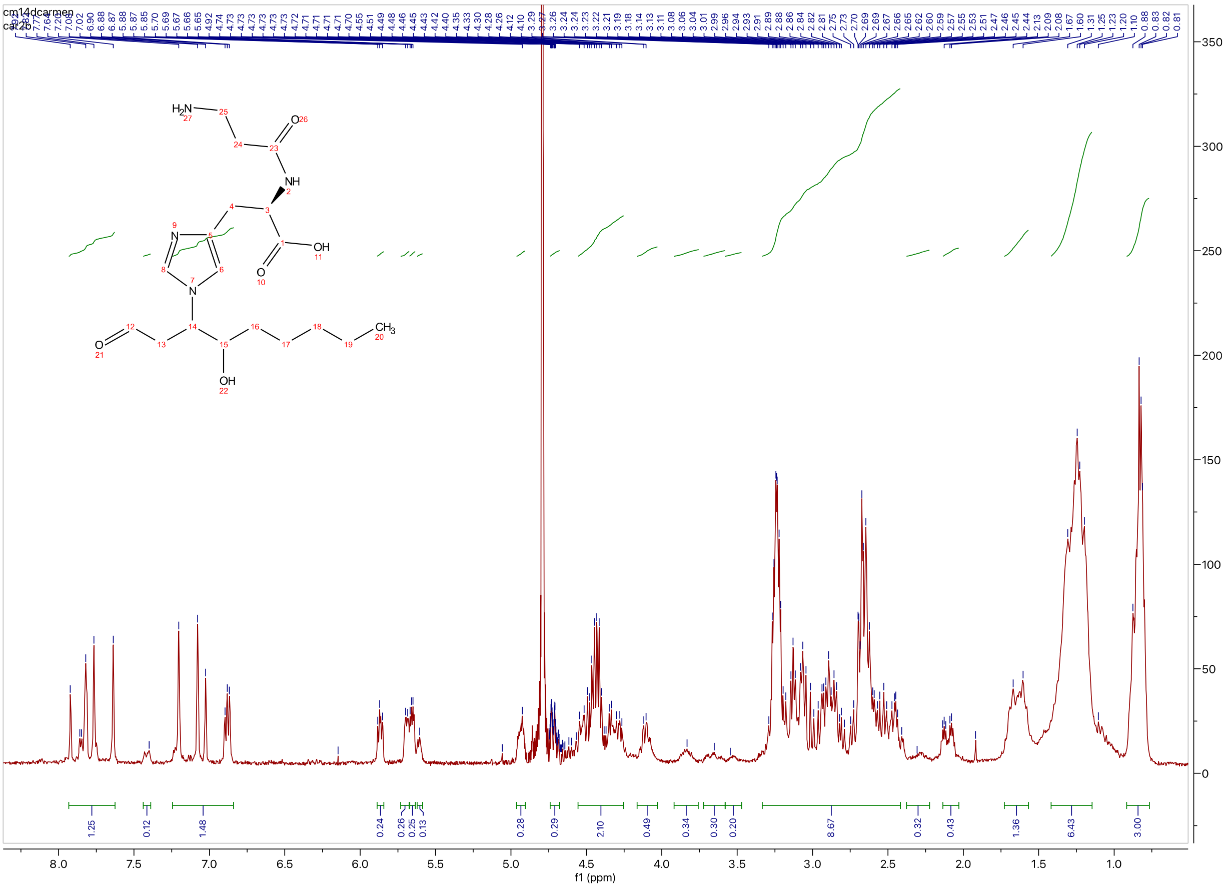

Figure S2 – ^{1}H NMR spectrum of CAR-HNE.

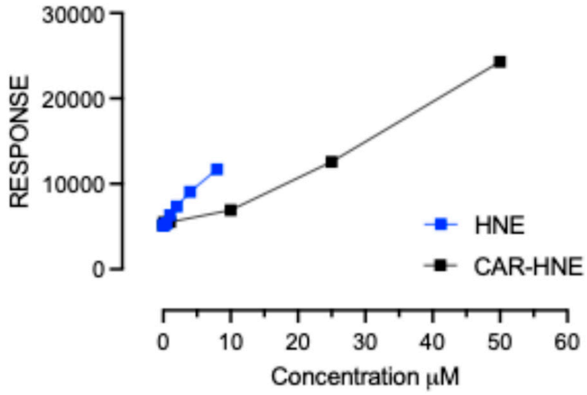

Figure S3 – Dose-dependent activation of Nfr2 induced by CAR-HNE and HNE as determined by the PathHunter Enzyme Fragment Complementation Assay Platform.

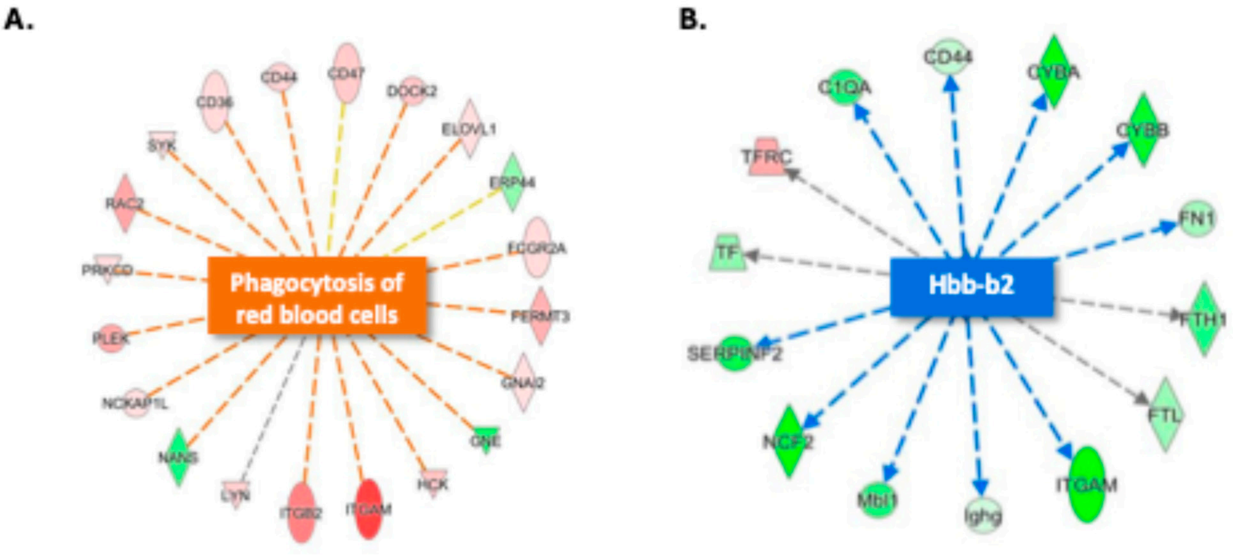

Figure S4 – Treatment with CAR-HNE suppressed heme-related signaling networks, including the Hbb-b2–centered interaction hub suggesting a mitigation of sustained inflammatory damage.
